# Radiation attenuation properties of chemically prepared MgO nanoparticles/HDPE composites

**DOI:** 10.1038/s41598-023-37088-y

**Published:** 2023-06-19

**Authors:** Ahmed M. El-Khatib, Mona M. Gouda, Mohamed S. Fouad, Mohamed Abd-Elzaher, Wegdan Ramadan

**Affiliations:** 1 Physics Department, Faculty of Science, Alexandria University, 21511 Alexandria, Egypt; 2 Department of Basic and Applied Sciences, Faculty of Engineering, Arab Academy for Science, Technology and Maritime Transport, Alexandria, Egypt

**Keywords:** Nanoscience and technology, Physics

## Abstract

Sheets of high-density polyethylene (HDPE) loaded with magnesium oxide in micro and nano were synthesized with different weight percentages of micro-MgO (0,5,10,20 and 30% by weight) and nano-MgO (5 and 30%) and shaped in form of disc and dog bone shape. The morphological, mechanical, and attenuation characteristics of each concentration were determined. The linear attenuation coefficients (LAC) of the prepared discs were calculated using a well-calibrated scintillation detector and five standard gamma-ray point sources (^241^Am, ^133^Ba, ^137^Cs, ^60^Co and ^152^Eu). The LAC was theoretically calculated for HDPE/micro-MgO composites using XCOM software. A good agreement between the theoretical and experimental results was observed. The comparison between micro and nano-MgO as a filler in HDPE was evaluated. The results proved that the loaded nano-MgO in different proportions of HDPE produced greater attenuation coefficients than its micro counterpart. The addition of nano MgO with different weight percentage led to a significant improvement in the mechanical properties of HDPE, the ultimate force and ultimate stress increased as the concentration of nano MgO increased, and the young modulus of HDPE also increased with increasing concentration of micro and nano MgO.

## Introduction

Protection of the public and workers against the hazards of radiation is a vital scientific problem. Exposure to radiation can harm living tissues by altering the structure of the cells and causing DNA damage. The type of radiation, its energy, and the total amount of radiation absorbed all influence the extent of the damage. Cellular damage may be healed, but certain cells may not recover as well as others and may develop cancer. As a result, the adoption of radiation safety regulations is required to limit and safeguard against radiation dangers^1,2^.

The growing utilization of radiation sources in many places, especially X-ray machines, makes the protection of the public and workers against their hazards a subject of interest. Shielding materials are required to attenuate radiation and protect personnel and other materials from harmful radiation such as X-rays, γ-rays, and neutrons. There are many materials widely used to attenuate radiation, which has many applications, such as lead, concrete, polymers, epoxy resin, colemanite, and glasses^3–8^ that are most commonly used in shielding materials for gamma and neutrons, but such materials may have some drawbacks, for example, variation in composition and water content that causes uncertainty in results^9–11^. The problem is serious, but the experimental work to determine the attenuation parameters needs a simple experimental setup. Most of the research concentrated on studying the attenuation of radiation by directing it toward the shielding material, whether these are organic substances, elements, or compounds^12^. New materials like nanomaterials may play an important role in exploring new shielding materials^13^ with higher attenuation parameters and higher stability than ordinary ones^14^. The scope of nanomaterials utilization is broad and currently, they are important in many diverse fields ranging from medicine, and the environment to clean sustainable energy^15,16^.

HDPE is more rigid than PE and is a good barrier to prevent moisture. HDPE remains in its solid state at a temperature lower than 134 °C, and does not emit harmful radiation^17,18^. In recent years, nanoparticles have attracted much attention because of their unique chemical and physical properties, like thermal conductivity and strength. In this context, MgO nanoparticles have high chemical and thermal stability, which makes it a good material for applications such as additives and paints. It is also widely used as a reinforcing reagent^19^. But most importantly, MgO is not toxic and poses no health threat when incorporated into composites. It is abundantly available in the earth’s crust and seawater which makes it eco-friendly as well as a good candidate when large-scale applications are intended.

The main target of this study was to use micro-sized magnesium oxide, nano-sized magnesium oxide, and HDPE to prepare composites in the form of sheets by the compression molding method to get experimental data for the attenuation of photons through these composites at different energies as well as changes in their mechanical properties.

## Materials and methods

### Samples preparation

**Figure 1:**
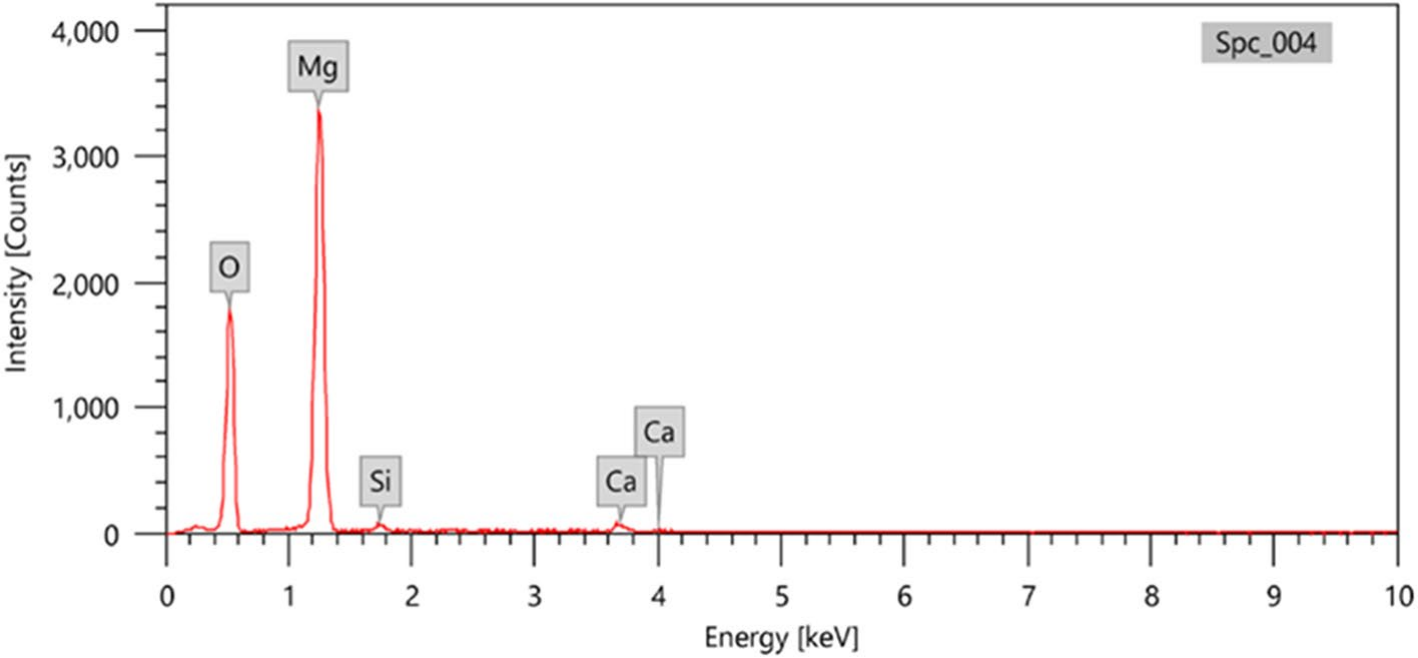
EDX analysis of Micro magnesium oxide.

A commercial HDPE as a matrix was obtained by Sidi Kerir Petrochemicals Company (sidpic), HD5403EAgrade with an index of melting flow point of 0.35 g/min and a density of 0.955 g/cm^3^. MgO microparticles in the powder phase were purchased from the Loba Company for laboratory reagents and fine chemicals (India) without further purification. EDX of the purchased MgO is shown in Fig. 1.

#### Mgo nanoparticles preparation

**Figure 2:**
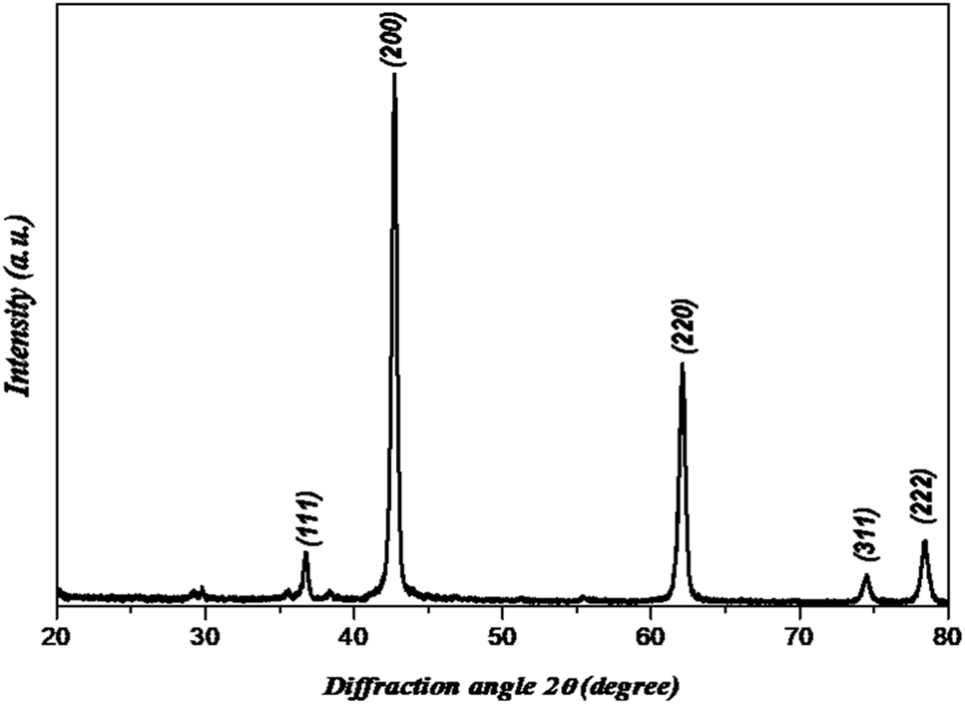
XRD of the synthesized MgO nanoparticles.

**Figure 3:**
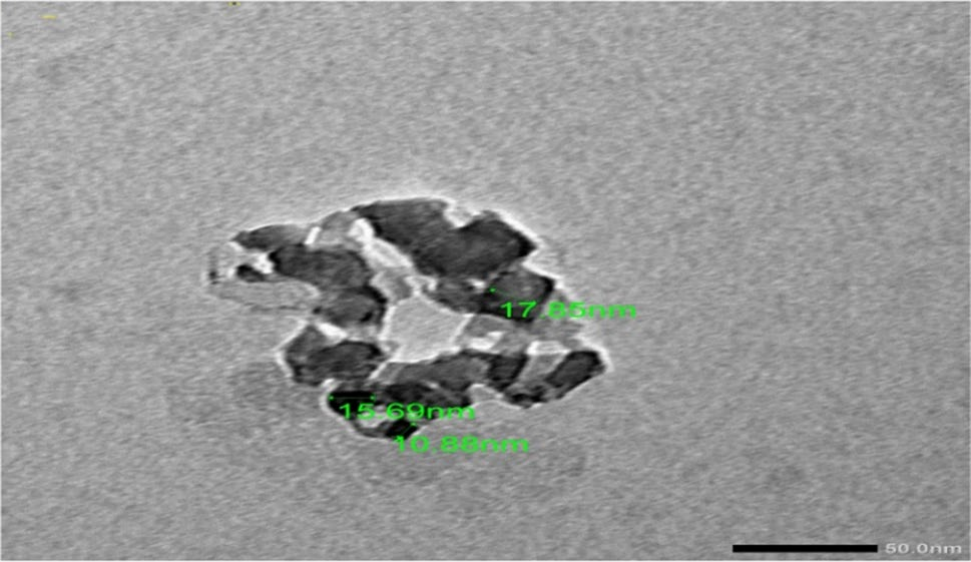
TEM image of Nano magnesium oxide.

**Figure 4:**
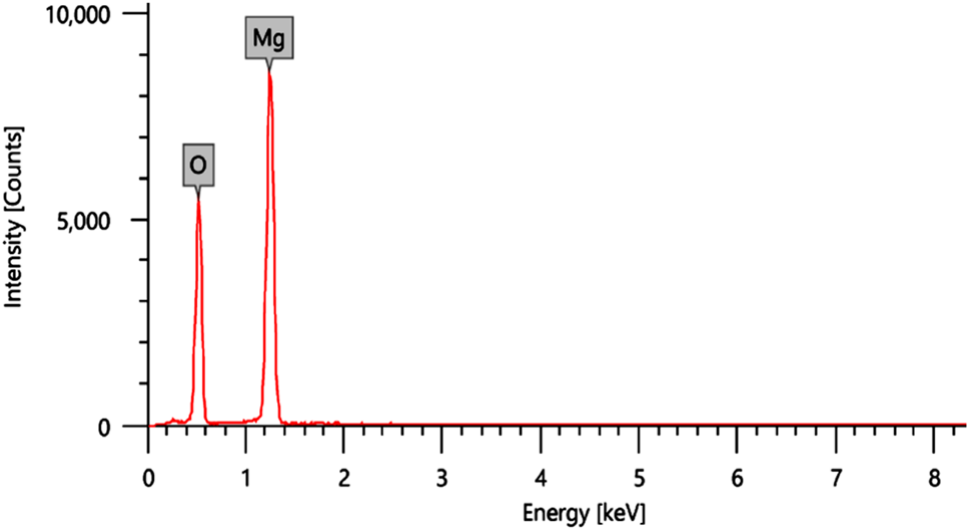
EDX analysis of Nano magnesium oxide.

Magnesium oxide nanoparticles were synthesized using magnesium nitrate (MgO_3_H_12_O_6_) as source material with sodium hydroxide and all chemicals were used without extra purification. For the typical experimental procedure, 0.2 M magnesium nitrate hydrated (MgO_3_H_12_O_6_) was dissolved in 100 ml of deionized water. A 0.5 M sodium hydroxide solution was added dropwise to the prepared magnesium nitrate solution while stirring it continuously. A white precipitate of magnesium hydroxide appeared in a beaker after a few minutes. The stirring continues for 30 min. The pH of the solution is 12 as measured by the pH paper. pH is one of the crucial parameters in determining the outcome of any synthesis process^20^. The precipitate was filtered and washed with methanol three times to remove ionic impurities. Samples were annealed at 100 °C for two hours, then the white powder undergoes a solid state reaction at a temperature of 500 °C for 4.5 h. The structure of the synthesized MgO nanoparticles was investigated using X-ray diffraction, XRD (X-ray Powder Diffraction-XRD-D2 Phaser, Bruker, Germany). Figure 2 shows the XRD pattern of MgO nanoparticles performed in *2θ* range from 20º to 80º. It shows major reflections at 2θ = 36.65°, 42.94°, 62.09°, 74.49° and 78.44° corresponding to (111), (200), (220), (311) and (222) planes of MgO and in good agreement with the standard JCPDS card (No: 78-0430). The mean crystallite size (D) of MgO nanoparticles was calculated for (2 0 0) and (2 2 0) planes using Scherrer's formula^21^ which is given by Eq. (1).

1 $$D = k\lambda /\beta \cos \theta$$

where ‘k’ is the shape factor (0.90), ‘λ’ is the wavelength of X-ray target (Cu Kα radiation), ‘β’ is the full width at half maximum (FWHM), and ‘θ’ is the angle of reflection of the peak. The calculated particle size of MgO nanoparticles from the XRD peaks was found to be around 18 nm which is comparable to the observed value from TEM. On the other hand, shape and size of the prepared MgO nanoparticles were further investigated using TEM (JSM1400plus-JEOL) as shown in Fig. 3. To verify the accuracy of the preparation and that it is magnesium oxide, it was analyzed by EDX as shown in Fig. 4.

### Synthesis of composite sheets

**Figure 5:**
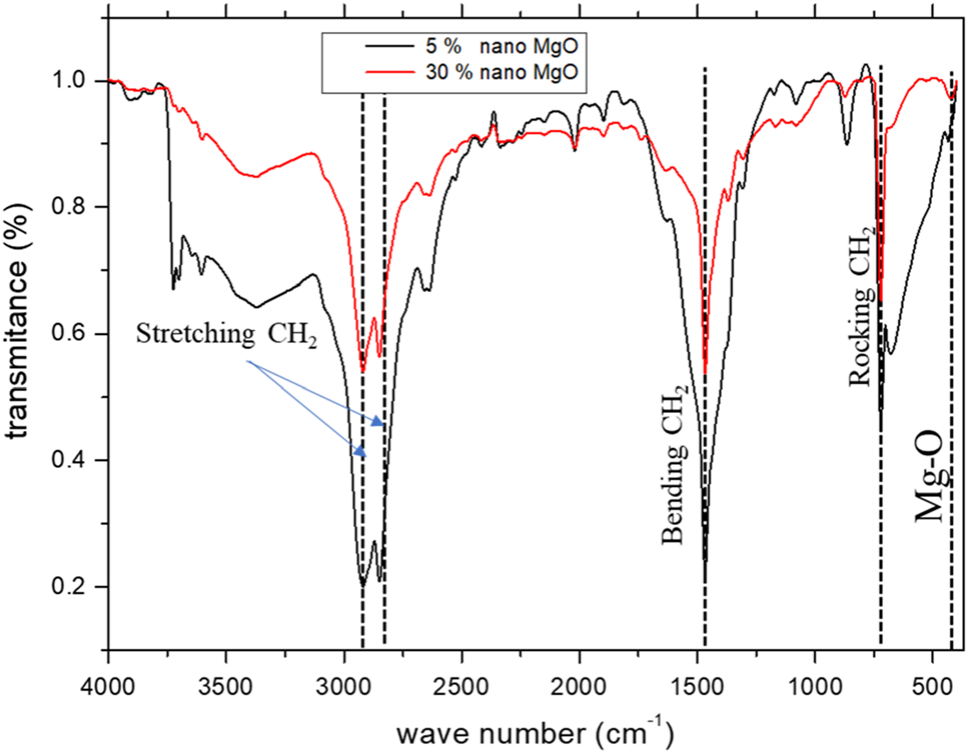
FTIR spectra of HDPE with 5wt% and 30wt% MgO nanoparticles.

The composites were prepared by using the compression molding method for the following samples (0 wt%, 5 wt%, 10 wt%, 20 wt% and 30 wt% ) of micro magnesium oxide/HDPE and (5wt% and 30wt% ) of Nano HDPE/MgO. Firstly, we used an electric balance of sensitivity 0.0001 g to weigh HDPE and fillers. In order to prepare the composite sheets, HDPE was placed between two roll mills at a temperature of 185 °C to make sure that it was completely melted (which is higher than the melting point of HDPE 134 °C) for 18 min with the rotation speed of 40 rpm. MgO powder was added gradually with continuous rolling for 10 min to obtain a uniform distribution of powder in HDPE. The completely mixed sample was placed in an iron frame of dimensions (12.5 × 12.5 × 0.3 cm^3^) then, the samples were compressed by the hydraulic press at a pressure 10 Mpa and a temperature 185 °C for 15 min, the pressure was raised gradually to 20 Mpa through 15 min. The sample was kept under pressure for 30 min to cool down gradually to a temperature of 50 °C then, the resultant sheets were cut into circular disks of a diameter of 8 cm for micro and 2.5 cm for Nano to do radiation investigation. For structural analysis of the composites and in order to confirm the presence of Mgo nanoparticles in the matrix of HDPE, FTIR (Bruker Tensor 37 FT-IR, Bruker, Germany) study was carried out. FTIR spectra of the 5wt% and 30wt% of Nano HDPE/MgO composite are shown in Fig. 5. Measurements were performed for wavenumbers between 4000 cm^−1^ and 420 cm^−1^. The HDPE vibrations are evident in each composite. The two peaks in the region between 2919 cm^−1^ and 2849 cm^−1^ represent the stretching vibration of -CH2 group. Peaks at 1467 cm^−1^ and 721 cm^−1^ represent the bending vibration and rocking deformation of the -CH2 group, respectively^22^. A small peak at 419 cm^−1^ was observed which is normally assigned to the stretching vibration of Mg-O^23,24^. It is interesting to note that Mg-O stretching vibration peak is more pronounced in the case of the composite containing 30 wt% of MgO when compared to that containing 5wt% MgO. However, this is as expected due to the increase in Mgo content in the composite.

### Instrumentations

#### Scanning electron microscope imaging

**Figure 6:**
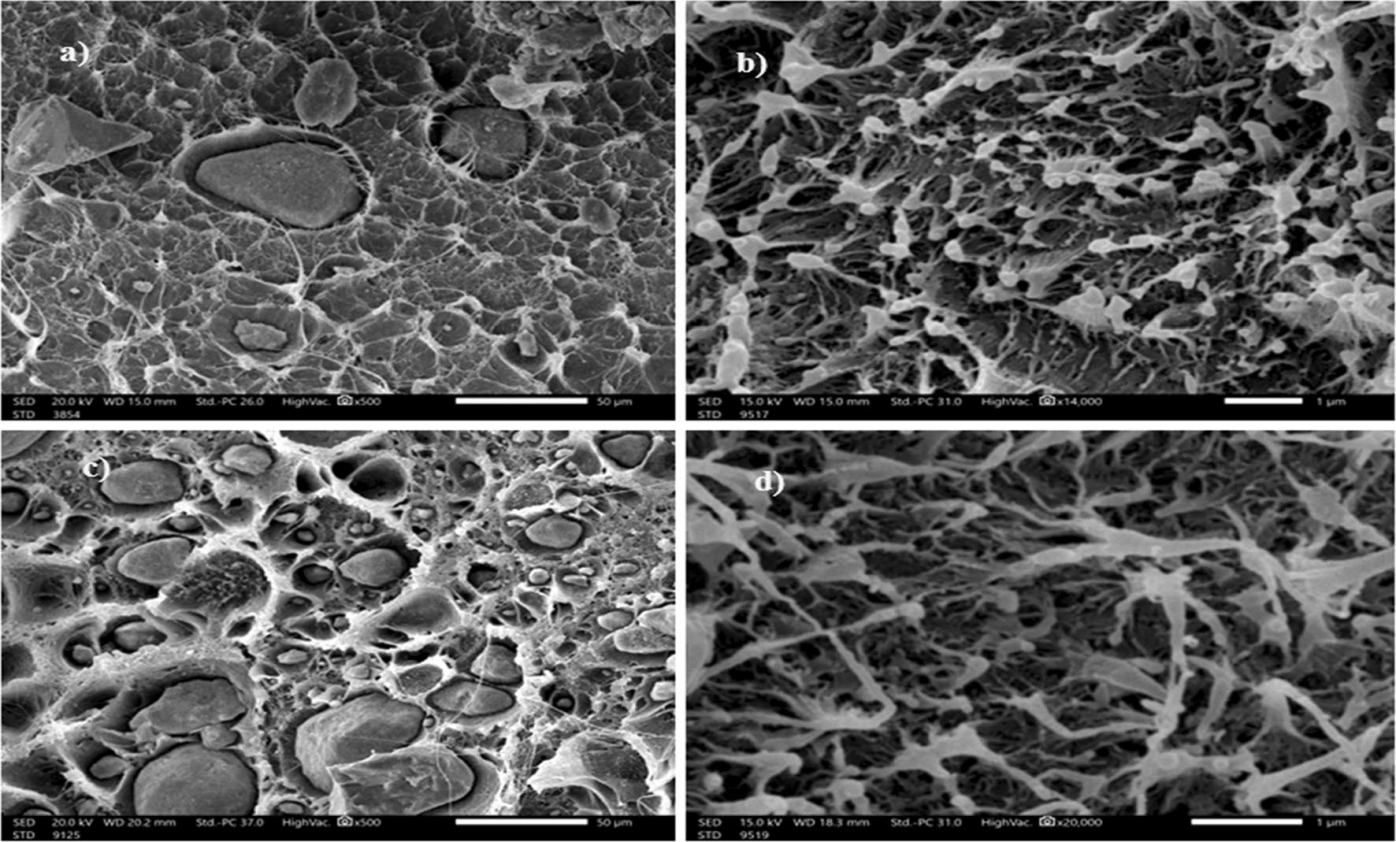
SEM images of (**a**) 5wt% micro MgO /HDPE, (**b**) 5wt% nano MgO /HDPE, (**c**) 30wt% micro MgO /HDPE and (**d**) 30wt% nano MgO /HDPE.

SEM or scanning electron microscope analysis (JSM-6010LV, JEOL) was used to observe the distribution as well as the shape of Micro and Nano MgO particles inside the matrix, in addition to the cross-section morphologies of the HDPE/MgO composite as shown in Fig. 6. The images were obtained from SEM at a magnification order of 5000 × at 20 kV.

#### Synthesis of sample for mechanical test

**Figure 7:**
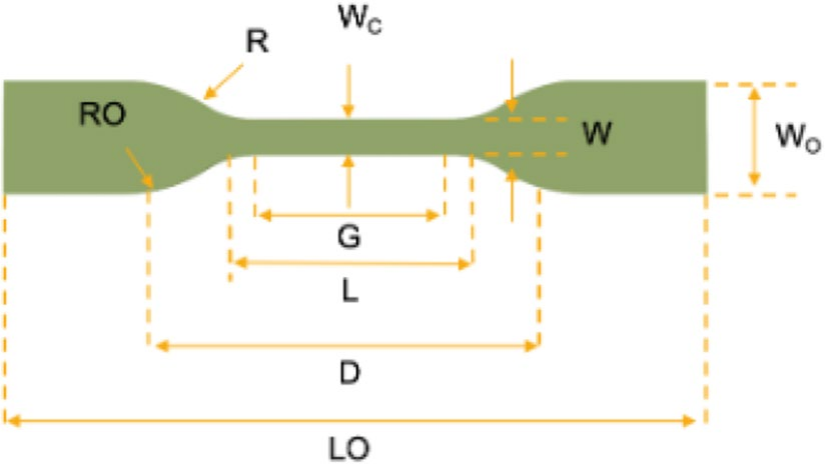
Shape of tensile test specimens according to ASTM D 638-IV(ASTM, 2014).

A mechanical testing machine (H10KS) was used for measuring ultimate stress and strain by applying stress reaches to 1.56 Mpa, the samples have dog bone–shaped according to the ASTM D 638-IV (ASTM,2014) with dimensions 6.2 mm in width, overall length 140 mm and 64 mm gauge length and 2.5 mm in thickness as shown in Fig. 7 and measuring the corresponding strain.

#### Radiation measurements setup

The attenuation of photons with different energies radiated from different standard radioactive point sources through the prepared composites was investigated by a gamma spectrometer. The sources were chosen to cover a wide range of energy where five radioactive sources were used as follows, Am-241 (0.06 MeV), Ba-133 (0.081, 0.356 MeV), Cs-137(0.662 MeV), Co-60(1.173, 1.333 MeV) and Eu-152(0.121, 0.244, 344 ,0.778, 1.085, 1.408 MeV). These radioactive point sources were purchased from the Physikalisch—Technische Bundesanstalt (PTB) in Braunschweig and Berlin, which is the national institute for Science and Technology and the highest technical authority of the Federal Republic of Germany for the field of metrology and certain sectors of safety engineering^25–30^.

**Figure 8:**
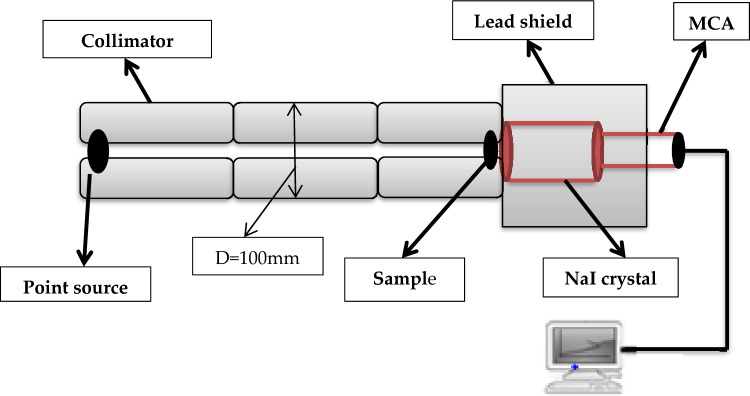
The experimental setup to measure the attenuation coefficient.

The spectrometer consists of NaI (Tl) cylindrical detector of dimension (3˝ × 3˝) with relative efficiency 15% at the energy of Cs-137 (0.662 MeV).) and a lead-collimator with an inner diameter of 8 mm and an outer diameter of 100 mm was used as a house shield for the radioactive source, composite material, and detector. Plus, the source was placed at 508.67 mm from the attenuating sheets while the sheets were placed straight forward on the detector to verify very good geometry. The illustration of the experimental setup shown in Fig. 8 was measured for all discussed HDPE/MgO samples using a good geometry technique of gamma-ray spectroscopy in the radiation physics laboratory, faculty of science, Alexandria University. The collected spectra were analyzed by using Genie 2000 software. The counting time in each run for the same sample with a certain thickness was limited by the value of the statistical error in the peak area for the considered energy to be less than 1%. The previous step was repeated for all composite samples with varying thicknesses and for all considered energies where the count rate (N) was determined from the peak area divided by the counting time.

### Shielding parameters

The count rate (N) was measured for all energies as a function of disc thickness (x) either for micro or nano MgO /HDPE samples. From the slope, LAC was calculated experimentally by the next equation^31,32^.

2 $${\text{LAC}} = \frac{1}{{\text{x}}} \ln \frac{{{\text{N}}_{0} }}{{\text{N}}}$$

To confirm the validity of LAC values, LAC for HDPE loaded with micro filler were obtained from the XCOM software^33,34^ compared with the experimental values. The relative deviation between the two values was calculated by:

3 $${\text{R}}\left( {\text{\% }} \right) = \frac{{{\text{LAC}}_{{{\text{xcom}}}} - {\text{ LAC}}_{{{\text{exp}}}} }}{{{\text{ LAC}}_{{{\text{exp}}}} }}{ } \times 100$$

while the relative increasing between the results of LAC of the micro and the results of nano filler evaluated by:

4 $${\text{R}}\left( {\text{\% }} \right) = \frac{{{\text{LAC}}_{{{\text{nano}}}} - {\text{ LAC}}_{{{\text{micro}}}} }}{{{\text{ LAC}}_{{{\text{micro}}}} }}{ } \times 100{ }$$

The other radiation attenuation parameters based on LAC calculations, such as half value layer (HVL), mean free path (MFP) and tenth value layer (TVL) were estimated from the following equations^35^.

5 $${\text{HVL}} = \frac{{{\text{ln}}2}}{{{\text{LAC}}}},\;{\text{MFP}} = \frac{1}{{{\text{LAC}}}},\;{\text{TVL}} = \frac{{{\text{ln}}10}}{{{\text{LAC}}}}$$

The effect of chemical composition of shielding material was elucidated using the effective atomic number (Z_eff_) which depends on the mass attenuation coefficient (µ/ρ) as shown in the following equation.

6 $$Z_{eff} = \frac{{\mathop \sum \nolimits_{i} w_{i} A_{i} \left( {\frac{\mu }{\rho }} \right)_{i} }}{{\mathop \sum \nolimits_{i} w_{i} \frac{{A_{i} }}{{Z_{i} }}\left( {\frac{\mu }{\rho }} \right)_{i} }}$$

where (µ/ρ)_i_ ,w_i_, A_i_ and Z_i_ are the mass attenuation coefficient , the weight fraction, the atomic weight and the atomic number respectively for constituent element in the sample. Additionally, the G-P fitting method^36^ was used to calculate the equivalent atomic number (Zeq), energy absorption buildup factors (EABF) and exposure buildup factors (EBF) for energy range from 0.015 MeV to 15 MeV.

7 $${\text{Zeq}} = \frac{{{\text{Z}}_{1} \left( {{\text{log R}}_{2} { } - {\text{ log R}}} \right){ } + {\text{ Z}}_{2} \left( {{\text{log R }} - {\text{ log R}}_{1} } \right){ }}}{{{\text{log R}}_{2} - {\text{log R}}_{1} }}$$

8 $${\text{P}} = \frac{{{\text{P}}_{1} \left( {{\text{log Z}}_{2} { } - {\text{ log Z}}_{eq} } \right){ } + {\text{ P}}_{2} \left( {{\text{log Z}}_{eq} { } - {\text{ log Z}}_{1} } \right){ }}}{{{\text{log Z}}_{2} - {\text{log Z}}_{1} }}$$

9 $${\text{B}}\left( {{\text{E}},{\text{x}}} \right) = {1} + \left( {{\text{b}} - {1}} \right)\left( {{\text{K}}_{{\text{x}}} - {1}} \right){\text{K}} - {1}\;{\text{For}}\;{\text{K}} \ne {1}$$

10 $${\text{B}}\left( {{\text{E}},{\text{x}}} \right) = {1} + \left( {{\text{b}} - {1}} \right){\text{x}}\;{\text{For}}\;{\text{K}} = {1}$$

11 $${\text{K}}\left( {{\text{E}},{\text{ x}}} \right) = {\text{ ca}}^{{\text{x}}} + {\text{d}}\frac{{{\text{tansh}}\left( {\frac{{\text{x}}}{{{\text{X}}_{{\text{k}}} }} - 2} \right) - {\text{tansh}}\left( { - 2} \right)}}{{1 - {\text{tansh}}\left( { - 2} \right)}}\;{\text{for}}\;{\text{x}} \le 40\;{\text{mfp}}$$

where R is defined as the ratio of mass attenuation coefficient by Compton effect and the total mass attenuation coefficient (μ_comp_/μ_total_) for given shielding material and for all the elements. Z_1_ and Z_2_ are the atomic numbers of elements that correspond to the ratios R_1_ and R_2_. The P stands for the G-P fitting parameters (b, c, a, X_k_ and d) are used for the calculation of EABF and EBF, while P_1_ and P_2_ are the values of G-P fitting parameters that correspond to the atomic numbers Z_1_ and Z_2_. In addition, E and x represent the incident photon energy and the mean free path's penetration depth, respectively.

## Results and discussion

### Mechanical results

**Figure 9:**
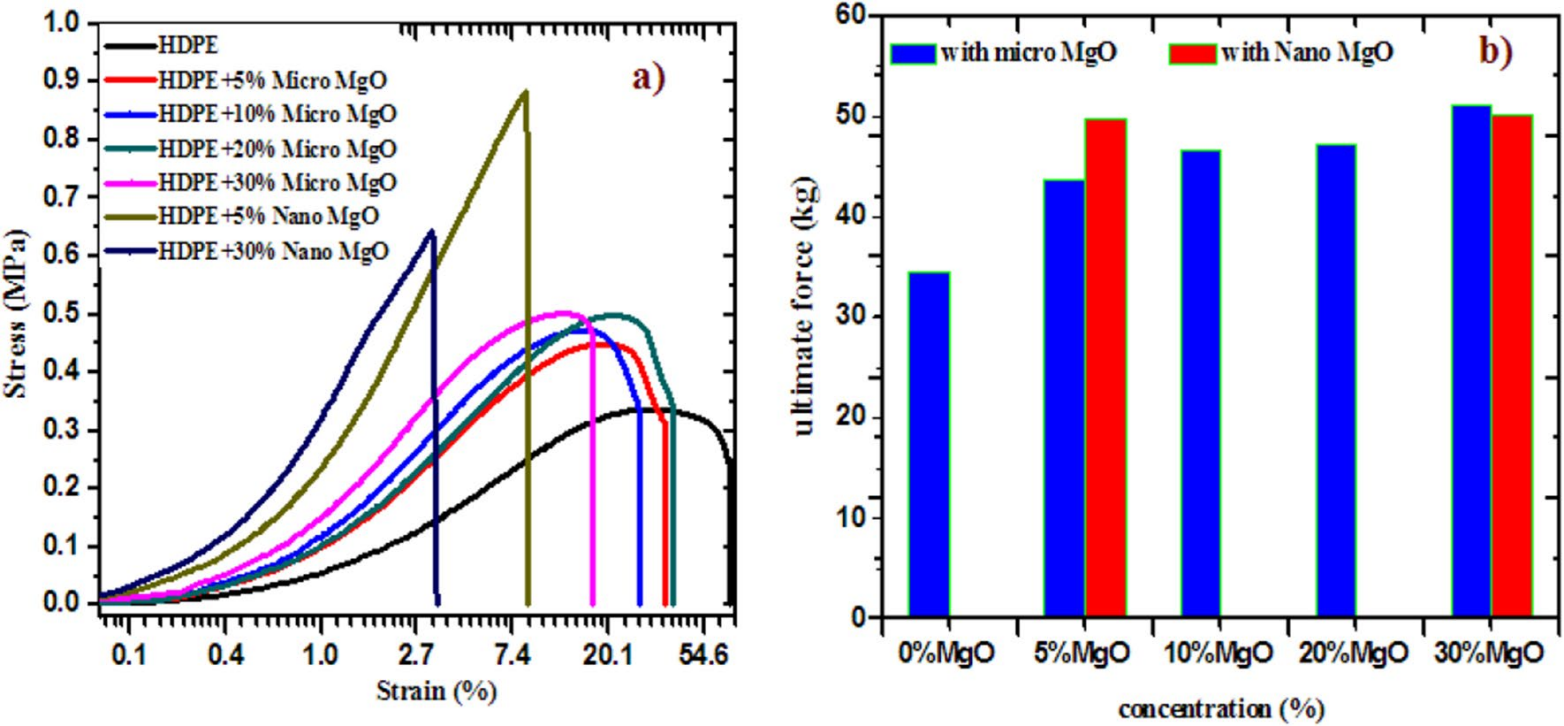
The mechanical results of polypropylene added with different sizes and concentrations of MgO (**a**) stress–strain curve, (**b**) ultimate force as a function of MgO concentration.

To investigate the change in the mechanical properties of the HDPE due to the addition of micro and nano MgO oxide in different percentages. The dog- shaped samples were subjected to gradually increasing stresses while the produced strains were recorded and the results are depicted as shown in Fig. 9. It is very clear from the obtained results that the addition of MgO in any size will increase remarkably the ultimate force of the composite samples this increase is obvious at the concentration 5% for added MgO either for micro or nano but the composite samples loaded with 5% percent nano MgO could bearing more stresses than the 5% percent micro MgO. This could be explained based on the size of the particles inside the sample, where particles with nano size could be distributed uniformly inside the matrix of the composite to produce a specimen with higher cross linking in the case of nano than micro. Moreover, from the obtained results, the ultimate force is nearly the same for samples loaded with MgO with higher concentrations than the 5% MgO, which means the mechanical stability of the sample with added MgO is still better than the pure MgO.

### Shielding results

**Figure 10:**
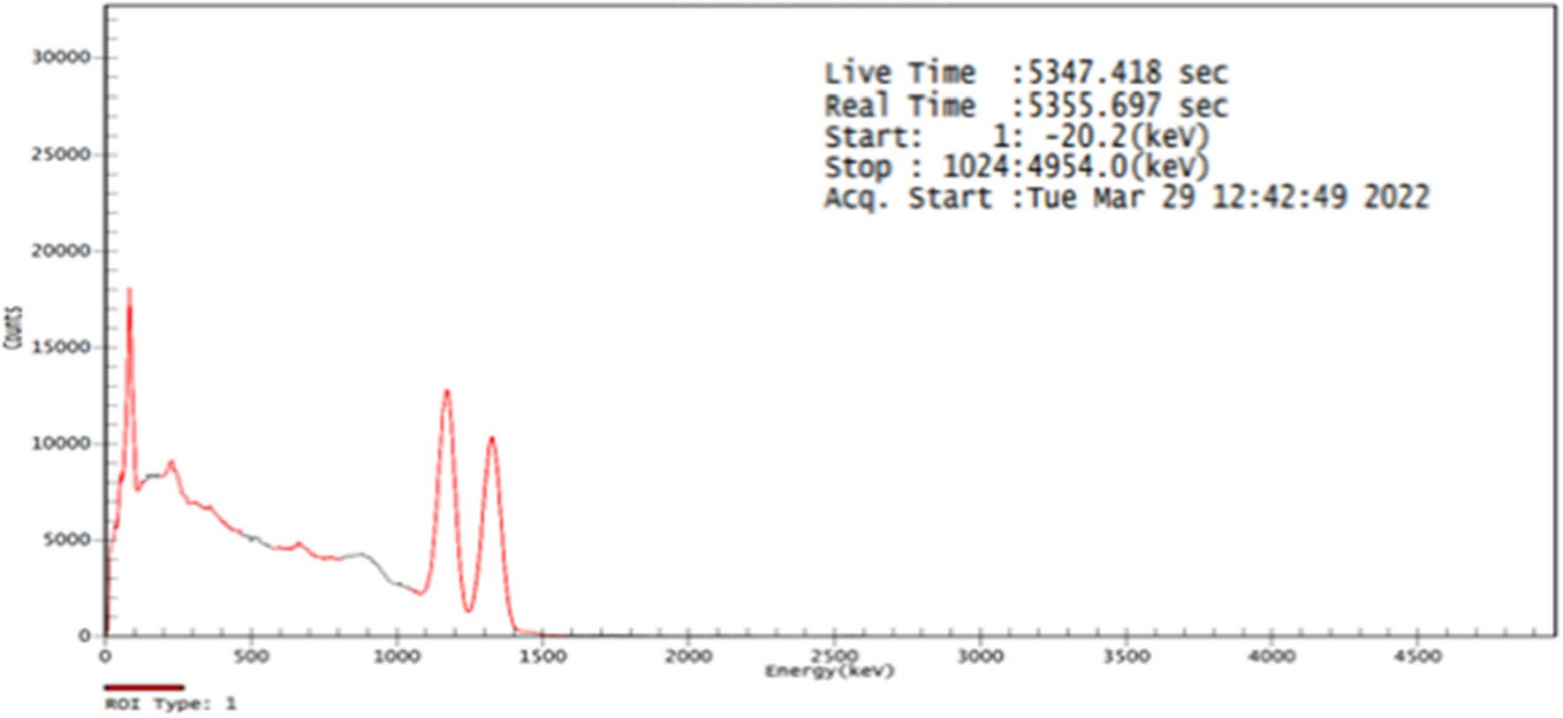
Spectrum of Co-60.

**Figure 11:**
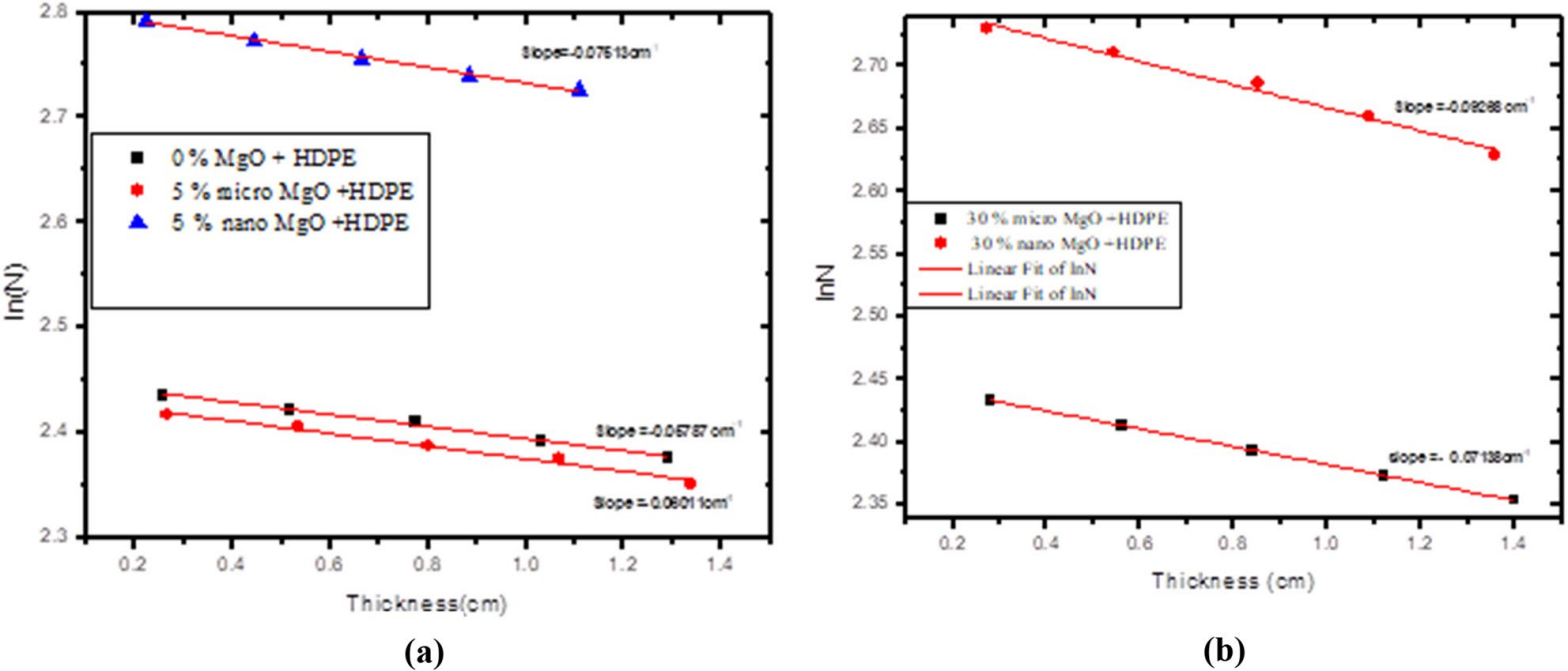
The Ln (count rate) as a function of thickness at 1.408 MeV energy for pure HDPE, 5% micro MgO, 5% nano MgO,30% micro MgO and 30% nano MgO.

The shielding properties of the prepared composites HDPE/ MgO were examined by using standard gamma point sources having energies between 0.06 and 0.1408 MeV, Fig. 10 shows the spectrum of Co-. The geometrical setup between the source, sample, and scintillation detector must verify the conditions of Beer-Lambert law. The calculated count rates (N) for HDPE samples loaded with micro MgO with concentrations (0,5,10,20 and 30% by weight) and nano-MgO with concentrations (5 and 30%) were determined by using software of Genie 2000 as function of sample thickness. All the data for all measurements were displayed on graphs where the Y-axis represents log(N) and X- axis represents the thickness X to get straight line, its slope =—linear attenuation coefficient µ (cm^−1^). Figure 11 depicts, for example, the curves for some HDPE samples loaded either with micro or nano MgO, where Table 1 gives all the shielding parameters for all samples and table (2) shows the relative increasing R(%) between the results of LAC of the micro and the results of nanofiller. It is clear from the example curves and the values of LAC mentioned in Tables 1 and 2 that the presence of MgO, either as micro or nano enhances the attenuation properties of HDPE at all used photon energies. Moreover, the attenuation of photons in nanocomposites was higher than that of micro composites at the same concentrations.

**Table 1:** Radiation parameters of prepared micro MgO and HDPE.

Sample	Energy (keV)	µ_EXP_ (cm^−1^)	µ_EXCOM_ (cm^−1^)	Half value layer (cm)	Tenth value layer (cm)	Density (g/cm^3^)	${\upmu }_{\mathrm{m}}$ EXP (cm^2^ g^−1^)	${\upmu }_{\mathrm{m}}$ Xcom (cm^2^ g^−1^)	∆%
HDPE + 5% MgO	59.33	0.1923	0.1945	3.6043	11.9733	1.03 ± 0.02	0.18750	0.1897	− 1.1513
	80.99	0.1864	0.1810	3.7178	12.3502		0.18180	0.1765	2.99846
	121.77	0.1608	0.1639	4.3095	14.3160		0.15680	0.1599	− 1.9196
	244.66	0.1300	0.1328	5.3319	17.7121		0.12680	0.1295	− 2.1163
	344.28	0.1129	0.1174	6.1394	20.3949		0.11008	0.11450	− 3.8554
	356.01	0.1125	0.1158	6.1624	20.4710		0.10967	0.11300	− 2.9415
	661.66	0.0875	0.0896	7.9207	26.3122		0.08532	0.08744	− 2.4745
	778.90	0.0814	0.0827	8.5121	28.2768		0.07940	0.08072	− 1.6352
	1085	0.0718	0.0710	9.6538	32.0694		0.07001	0.06932	0.99555
	1173.5	0.0660	0.0683	10.5022	34.8876		0.06435	0.06664	− 3.4293
	1332.33	0.0656	0.0640	10.5630	35.0896		0.06398	0.06242	2.5058
	1408.01	0.0620	0.0622	11.1797	37.1384		0.06045	0.06067	− 0.3553
HDPE + 10% MgO	59.33	0.2066	0.2073	3.3553	11.1462	1.09 ± 0.02	0.19001	0.1907	− 0.3587
	80.99	0.1913	0.1913	3.6233	12.0365		0.17590	0.1760	− 0.0201
	121.77	0.1759	0.1728	3.9407	13.0910		0.16178	0.1590	1.7237
	244.66	0.1412	0.1399	4.9089	16.3072		0.12988	0.1287	0.9085
	344.28	0.1238	0.1236	5.5971	18.5932		0.11391	0.1137	0.18583
	356.01	0.1232	0.1221	5.6266	18.6913		0.11331	0.1123	0.8946
	661.66	0.0964	0.0944	7.1918	23.8906		0.08865	0.08687	2.01139
	778.90	0.0833	0.0872	8.3220	27.6454		0.07661	0.08020	− 4.6825
	1085	0.0739	0.0748	9.3706	31.1286		0.06804	0.06887	− 1.2201
	1173.5	0.0721	0.0719	9.6110	31.9271		0.06633	0.06620	0.20818
	1332.33	0.06665	0.06742	10.3990	34.5474		0.06130	0.06202	− 1.163
	1408.01	0.06537	0.06553	10.6034	35.2238		0.06012	0.06028	− 0.250
HDPE + 20% MgO	59.33	0.2175	0.21475	3.1860	10.5836	1.12 ± 0.03	0.19501	0.1925	1.28913
	80.99	0.1954	0.19545	3.5473	11.7839		0.17515	0.1752	− 0.0279
	121.77	0.1748	0.17537	3.9633	13.1659		0.15676	0.1572	− 0.2768
	244.66	0.1418	0.14168	4.8860	16.2359		0.12712	0.1270	0.0943
	344.28	0.1251	0.12517	5.5370	18.3941		0.11220	0.1122	0.0068
	356.01	0.123	0.12361	5.6350	18.7202		0.11025	0.1108	− 0.4952
	661.66	0.0956	0.09563	7.2480	24.0780		0.08571	0.08572	− 0.0009
	778.90	0.0877	0.08828	7.8973	26.2343		0.07861	0.07914	− 0.672
	1085	0.0750	0.07580	9.2370	30.6847		0.06726	0.06795	− 1.0198
	1173.5	0.0785	0.07288	8.8260	29.3211		0.07039	0.06533	7.19115
	1332.33	0.0690	0.06827	10.0450	33.3708		0.06185	0.06120	1.05093
	1408.01	0.0656	0.06636	10.5590	35.0789		0.05883	0.05949	− 1.10813
HDPE + 30% MgO	59.33	0.2348	0.2348	2.9510	9.8066	1.21 ± 0.03	0.19440	0.1944	0
	80.99	0.21031	0.2106	3.2950	10.9485		0.17408	0.1744	− 0.18
	121.77	0.1908	0.1878	3.6320	12.0680		0.15793	0.1555	1.54
	244.66	0.15899	0.1513	4.3580	14.4825		0.13160	0.1253	4.789
	344.28	0.13073	0.1337	5.3010	17.6132		0.10821	0.1107	− 2.29
	356.01	0.12555	0.1320	5.5190	18.3399		0.10390	0.1093	− 0.5197
	661.66	0.09822	0.1020	7.0550	23.4431		0.08130	0.08458	4.03
	778.90	0.0979	0.09432	7.07865	23.5197		0.08103	0.07808	3.64
	1085	0.0819	0.08099	8.4615	28.1145		0.06779	0.06704	1.1098
	1173.5	0.07608	0.07786	9.1088	30.2653		0.06307	0.06445	− 2.18
	1332.33	0.0716	0.0729	9.6787	32.1590		0.06026	0.06039	− 0.0019
	1408.01	0.07125	0.0709	9.7263	32.3169		0.0589	0.05870	0.004

**Table 2:** The relative increasing R(%) between the results of LAC of the micro and the results of nano filler.

Energy(keV)	LAC of (5% nano MgO)	LAC of (5% micro MgO)	R (%)	LAC of (30% nano MgO)	LAC of (30% micro MgO)	R (%)
59.33	0.32621	0.19231	69.6	0.4453	0.2348	89.6
80.99	0.2951	0.18644	58.3	0.34843	0.21031	65.6
121.77	0.25096	0.16084	56.0	0.30412	0.1908	59.4
356.01	0.16046	0.11248	42.6	0.19347	0.12555	54.1
661.66	0.12271	0.08751	40.2	0.1484	0.09822	51.1
1173.5	0.09125	0.066	38.2	0.11165	0.07608	46.7
1332.5	0.08563	0.06562	30.5	0.09642	0.0716	34.7
1408.01	0.07409	0.062	19.5	0.09307	0.07125	30.6

**Figure 12:**
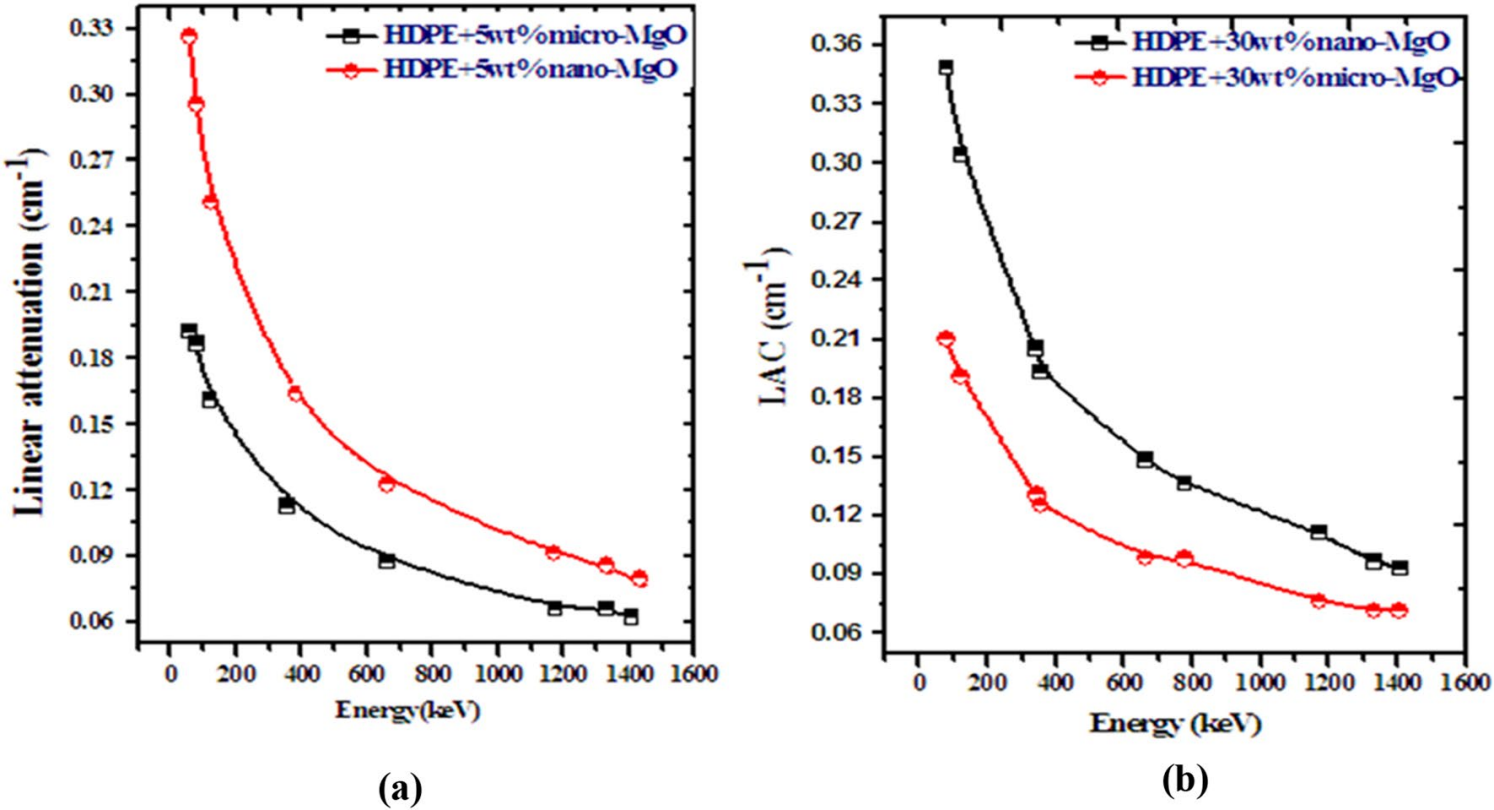
Linear attenuation coefficient comparison between micro and nano filler (**a**) 5% MgO and (**b**) 30% MgO.

A good question here is: why are these changes in the attenuation parameters? It is well known that the attenuation of photons inside any absorbing medium depends on the density, the atomic number of the medium, and the energy of the incident photons. Of course, the presence of MgO will increase both the density and the effective atomic number of the used composite in this work, which means that the electronic density of the absorbing medium will increase, and this in turn will increase the probabilities of interactions between the transmitting photons and the electrons. More interactions yield more energy loss and more attenuation. It is worthwhile mentioning that the distribution of nano particles inside the composite will help to increase these interactions and attenuation probabilities see Fig. 12. This illustration was confirmed by increasing the concentration of MgO either in micro or nano sizes where LAC values increase at any specific energy than those of pure HDPE. On the other hand, photon energy plays an important role in affecting the attenuation of photons where higher energies produce less attenuation because the Compton scattering within the range of the used photon energy in this work is predominant.

**Figure 13:**
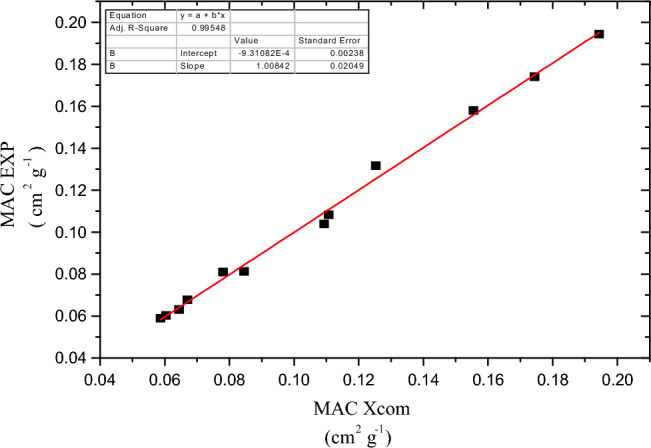
Relation between the experimental values of MAC and those calculated from XCOM program composites loaded with 30% micro MgO.

**Figure 14:**
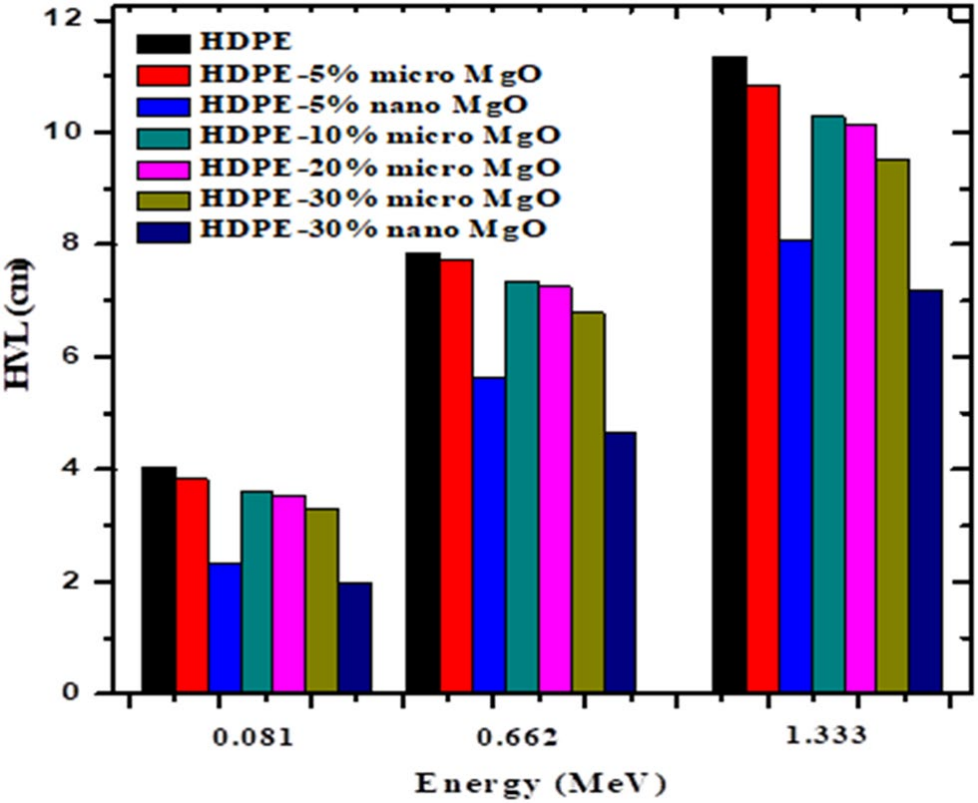
The HVL of the prepared samples at 0.081, 0.662 and 1.333 MeV.

Figure 13 displays the relation between the experimental values of LAC and those calculated from XCOM program composites loaded with micro MgO to see how true the experimental measurements are. An improvement in ability of composites of HDPE/MgO to attenuate gamma photons as shown in Fig. 14, at small energies range HVL decrease as the concentration of micro MgO filler increase, by replacing the micro MgO filler by nano MgO there was an additional improvement in radiation properties of the composite at energy of 0.08099 MeV. The value of HVL is 4.033 cm for a 5% micro HDPE/MgO composite and decreases to 1.989 cm in the case of a 30% nano HDPE/MgO composite, but in the case of w 5% nano HDPE/MgO composite, the value of HVL decreases to 2.349 cm. By increasing the energy of the incident gamma photon, we notice an increase in the thickness of material that is required to attenuate gamma radiation. At an energy of 1.332 MeV, the value of HVL decreases from 11.356 cm in the case of pure HDPE to 10.827 cm in the case of a 5% micro-HDPE/MgO composite and decreases to 8.0995 cm in the case of a 5% nano HDPE/MgO. By increasing the concentration of micro filler to 30%, HVL decreases to 9.508 cm and this value decreases to 7.189 cm in the case of 30% nano HDPE/MgO composite.

**Figure 15:**
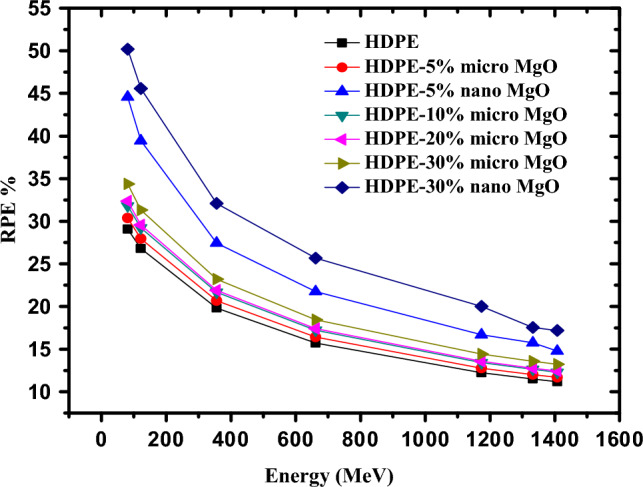
The RPE of the prepared samples as a function of photon energy.

Figure 15 shows that the radiation protection efficiency (RPE) which measures the effectiveness of shielding material for radiation protection and can be calculated by using Eq. (12) is a function of the energy of gamma ray photon and also depends on the concentration of micro and nano sized MgO in the HDPE matrix. At an energy of 0.08 MeV, RPE increases from 30.37% in the case of HDPE/ 5% micro MgO to 44.58% in the case of HDPE/ 5% nano MgO. By increasing the concentration of nano MgO in HDPE matrix to 30%, RPE increased to 50.19%. By increasing the energy of incident photon to 0.661 MeV, the value of RPE increased from 20.69% in the case of 5% micro MgO to 21.76 in the case of 5%nano MgO but at 30% concentration of nano MgO, the value of RPE is 25.68% at high energy 1.408 MeV and the value of RPE increased from 11.70% in the case of 5% micro MgO to 13.77% in the case of 5%nano MgO. By increasing the concentration of nano MgO to 30% , the value of RPE increased to 16.98%.

12 $${\text{RPE }} = \left( {1 - \frac{{\text{I}}}{{{\text{I}}0}}} \right) \times {1}00$$

**Figure 16:**
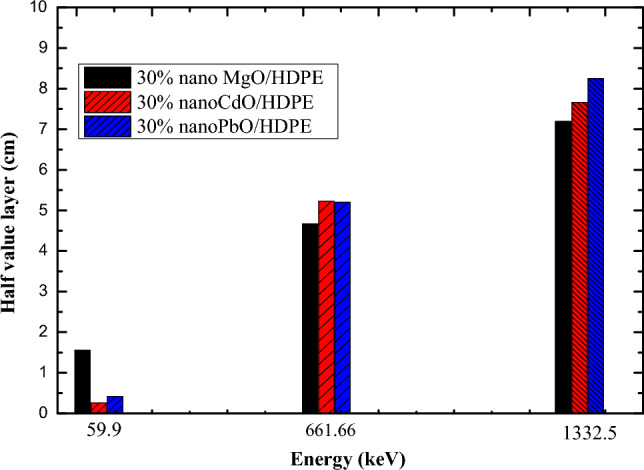
HVL of nano HDPE/MgO and HDPE/CdO and HDPE/PbO.

At low energy range as shown in Fig. 16, HDPE/PbO NPs composite of concentration 30% gives radiation shielding properties better than nano HDPE/CdO and nano HDPE/ MgO composite of the same filler concentration at energy 0.059 MeV. The value of HVL of HDPE/ PbO is 0.155658 cm and 0.25472 cm in the case of HDPE/CdO composite and 0.4182 cm in the case of HDPE/ MgO composite, but at medium and high energy range nano MgO/HDPE composite attenuate the gamma photon in better way than HDPE /CdO composite and HDPE/ PbO composite. At energy 0.661 MeV the value of HVL of HDPE/ PbO is 5.2042 cm and 5.22657 cm in the case of HDPE/CdO composite and 4.6708 cm HDPE/ MgO. At energy 1.332 MeV, the value of HVL of HDPE/ PbO is 8.2487 cm and 7.65739 cm in the case of HDPE/CdO and 7.18883 cm HDPE/ MgO.

**Figure 17:**
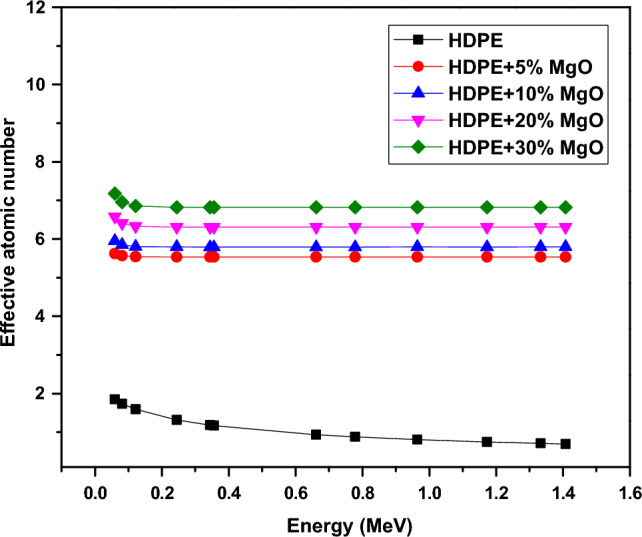
The effective atomic number (Z_eff_) variation with the incident gamma photons.

Figure 17 displays the effective atomic numbers (Z_eff_) which are calculated by Eq. (6) at gamma-ray energies ranging from 59.3 to 1408.01 keV. It is clearly seen that, the values of Z_eff_ for all samples vary with the range of atomic numbers, and the Z_eff_ values of each sample decrease with increasing gamma ray energy. Also, it has been found that, the values of Z_eff_ rise as the MgO content in HDPE compositions rises. The interactions with gamma rays in composite materials depend on their Z_eff_ value and photon energy.

**Figure 18:**
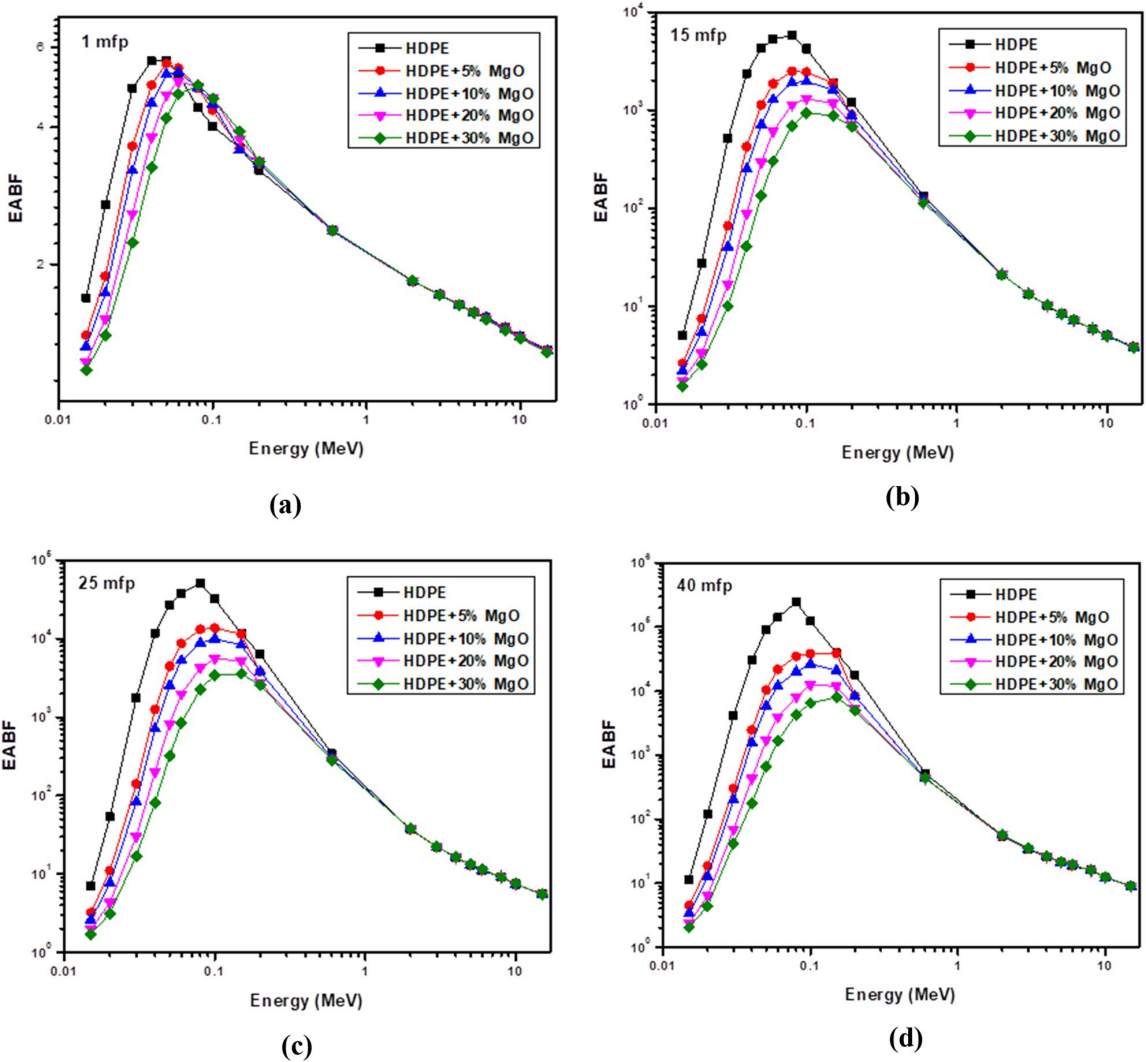
Variation of the energy absorption buildup factor (EABF) with photon energy at penetration depths of (**a**) 1 mfp, (**b**) 10 mfp, and (**c**) 25 mfp and (**d**) 40 mfp.

**Figure 19:**
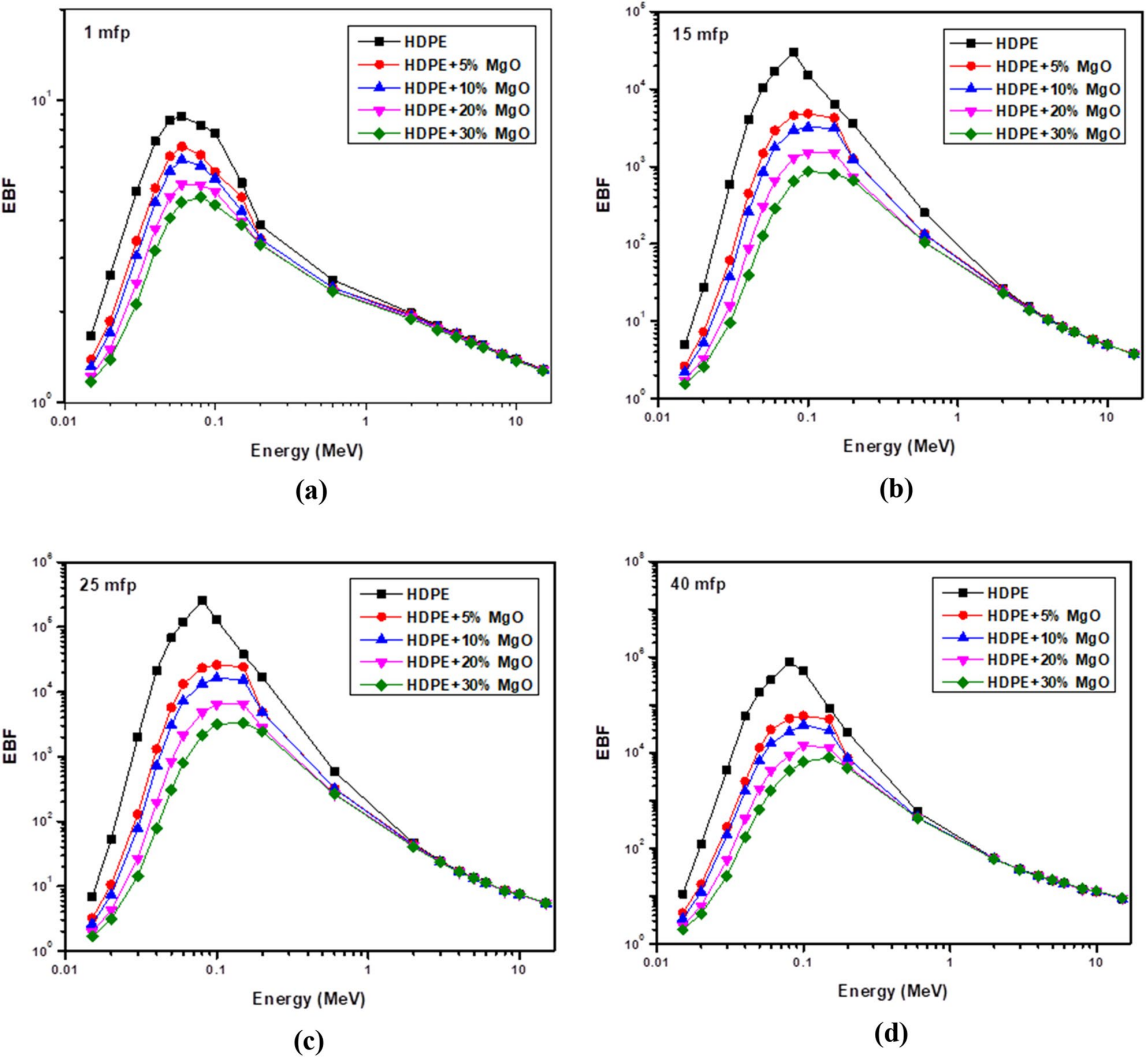
Variation of the exposure buildup factor (EBF) with photon energy at penetration depths of (**a**) 1 mfp, (**b**) 10 mfp, and (**c**) 25 mfp and (**d**) 40 mfp.

The energy absorption buildup factor (EABF) and exposure buildup factor (EBF) versus photon energy are shown in Figs. 18 and 19. EABFs and EBFs typically exhibit low values at low and high energies and high values at medium energies. In the studied energy range, photon interactions can be used to explain these corollaries. The photons are mostly annihilated or lose energy at low energy levels because the photoelectric effect is the predominant process. There is less photon buildup as a result. Then, all samples' EABFs and EBFs gradually increased. At medium energy, Compton scattering has a decisive role. As a result, secondary photon creation is improved, resulting in greater EABF and EBF. The photons disappear at high energies or have much reduced energy, just as they do at low energies. As a result, data for EBF and EABF show a declining trend after 1 MeV. Also, both buildup factors are significantly decreased by raising Mgo in the HDPE composition. Additionally, it is clear that, as the mean free path increases, the EABF and EBF values for all samples increase gradually. This observation stems from the fact that an increase in penetration depth causes the scattering volume to rise. Multiple scattering events occur in these conditions, resulting in a large number of scattered photons, which in turn causes the values of the buildup factors to climb.

## Conclusion

In this investigation, the effect of particle size and weight percentage of micro and nano MgO was determined by measuring the linear and mass attenuation coefficient of the HDPE/MgO composite at different photon energies. The compression molding technique was used to fabricate composites, and the morphological properties of composites were investigated by SEM. From SEM images, the distribution of nano MgO in the matrix is more uniform than micro MgO and also shows strong adhesion between the HDPE matrix and MgO. Adding MgO to the HDPE matrix led to a significant improvement in the mechanical and radiation properties of the composite. The ultimate force increased by increasing the concentration of micro MgO in the HDPE matrix led to an increase in the required force for the material to reach its ultimate point. Replacing micro MgO filler with nanofiller with low concentration led to additional improvement in the mechanical properties of the composite. RPE of composite also improved as the concentration of micro MgO increased, the mass attenuation coefficient increased at the same energy, and the HVL decreased significantly, and the ability of the HDPE/MgO composite improved by replacing micro MgO.

## Data Availability

All data generated or analyzed during this study are included in this published article.
